# Why deinstitutionalisation still matters: Reclaiming a contested horizon in global mental health

**DOI:** 10.1371/journal.pmen.0000699

**Published:** 2026-09-02

**Authors:** Cristian Montenegro, Felipe Szabzon, Sofía Bowen, Delia da Mosto

**Affiliations:** 1 Department of Global Health and Social Medicine, King’s College London, London, United Kingdom; 2 Instituto de Medicina Social, Universidade do Estado do Rio de Janeiro, Rio de Janeiro, Brazil; 3 Escuela de Salud Pública, Universidad de Chile, Santiago, Chile; PLOS: Public Library of Science, UNITED KINGDOM OF GREAT BRITAIN AND NORTHERN IRELAND

## Abstract

Psychiatric deinstitutionalisation (PDI) has re-emerged as an important concern in global mental health in response to the persistence of long-term institutionalisation and human rights abuses in institutions. However, contemporary responses continue to frame reform largely in managerial and technocratic terms, prioritising service coverage, bed reductions, workforce expansion, and scalability. We argue that this framing narrows the meaning of reform and its capacity to address the institutional, political, and social conditions through which psychiatric care is organised. Drawing on critical perspectives on PDI and experiences from South America, we show how PDI has developed through distinct relationships between institutional transformation, democratisation, social mobilisation, and citizenship. These trajectories illuminate contemporary dilemmas such as transinstitutionalisation, fragmented systems of care, and the persistence of segregationist and custodial logics within community settings. We propose three lenses for re-engaging with PDI as an ongoing political and ethical project: attending to context and history, decentring dominant geographies of reform, and foregrounding lived experience. Together, these lenses shift attention towards the sites where reform is actually produced—across research, policy, training, activism, and governance— and highlight the contribution of South American experiences to ongoing debates on institutional transformation.

## Introduction: The narrowing of reform

Psychiatric Deinstitutionalisation (PDI) is widely recognised as a cornerstone transformation in twentieth-century mental health care. It broadly refers to the reduction and closure of large, specialised psychiatric hospitals and the parallel development of comprehensive, community-based residential and treatment alternatives. This shift is often driven by the goal of improving quality of care and expanding rights for people with psychosocial disabilities while challenging traditional roles and practices within mental health care. Historically, the shift emerged from a radical critique of ‘asylums’, perceived as “total institutions” [1], and of institutional psychiatry, viewed as a system organised around the denial of autonomy and citizenship and as a tool of social control and marginalisation [2]. Guided by ethical and political values, reform challenged confinement and segregation as default responses to severe mental distress.

By the late 1980s, however, PDI had begun to lose much of its political and ideological edge in North America and Western Europe. In the USA, reform often took the form of dehospitalisation—understood less as a comprehensive transformation of care systems than as the reduction of long-stay beds and the discharge of patients from psychiatric hospitals [3]. While PDI had originally implied a coordinated shift towards community-based care, including the development of accessible services, social support systems, and alternative residential arrangements, in practice these elements were unevenly realised [4]. Community infrastructures were frequently underdeveloped or fragmented, and responsibility for care was redistributed to families without adequate support. As a result, many individuals experienced discontinuity of care, exposure to new forms of vulnerability, and, in some cases, re-institutionalisation in other settings, while logics of coercion and control persisted in modified forms [5,6]. Rather than a reconfiguration of the relationship between care, community, and citizenship, reform often resulted in a partial displacement of institutional functions.

In parts of Europe, early reform agendas, initially grounded in critiques of institutional psychiatry and oriented towards social inclusion and rights, were progressively reshaped by neoliberal governance and austerity measures [7–9] Policy priorities shifted towards efficiency, fiscal restraint, and accountability, with increasing emphasis on cost containment and service performance. Across both contexts, these developments were accompanied by a redefinition of how deinstitutionalisation was evaluated and governed, with an emphasis on bed numbers, shorter lengths of stay, and lower rates of hospitalisation, rather than the transformation of lived conditions and social relations of care [10,11]. Reorganised around managerial and financial logics, new treatment modalities frequently overlooked the social conditions necessary for recovery. At the same time, the growing centrality of risk management reframed patients as objects of surveillance and control, displacing earlier concerns with subjectivity and social integration [12,13]. This reorientation narrowed the meaning of reform itself, privileging what could be measured, managed, and scaled, and laying the groundwork for the technocratic framings that continue to shape global mental health today.

Yet this trajectory neither exhausted nor defined the possibilities of psychiatric reform elsewhere. In several countries of the Global South, and particularly in South America, psychiatric reform unfolded in close articulation with processes of democratisation, social mobilisation, and the development of distinct traditions within social medicine. These trajectories illustrate different ways in which deinstitutionalisation became entangled with parallel, intersecting projects of political and institutional transformation.

In Brazil, the rise of the *Movimento de Luta Antimanicomial* in the late 1980s transformed psychiatric reform into a broad-based political project, linking the critique of the asylum to struggles for citizenship, rights, and the construction of a universal public health system [14–16]. Emerging in opposition to the “madness industry” consolidated under dictatorship—characterised by the expansion of private psychiatric hospitals, overcrowding, and systematic neglect [17,18], the movement redefined PDI as a process of deconstruction of the asylum logic [19]. This approach unfolded across multiple dimensions, including legal-political struggles for rights, the development of territorial psychosocial services such as CAPS [Centros de Atenção Psicossocial], and broader sociocultural efforts to transform public understandings of madness [20,21].

In Argentina, reform trajectories were more discontinuous, shaped by cycles of innovation and political repression [22]. Mid-twentieth-century efforts to move beyond asylum-based care, including the development of psychiatric services in general hospitals and experiments with therapeutic communities, were interrupted by state violence during the 1976–1983 dictatorship, which targeted mental health professionals and reinstated custodial and biomedical approaches to care [23]. Following the return to democracy, reforms regained momentum, finding expression in provincial initiatives such as Río Negro’s Law 2440 and the 2010 National Mental Health Law, which established a rights-based framework oriented towards deinstitutionalisation and the integration of care into general hospitals [24,25].

In Chile, reform followed a different trajectory, marked by early attempts to radically reconfigure psychiatry through community-based and politically embedded approaches, followed by authoritarian interruption and later technocratic reconfiguration. In the late 1960s and early 1970s, pioneering epidemiological studies [26] and initiatives such as intracommunity psychiatry sought to shift mental health care beyond asylums and towards collective, socially grounded approaches to care, closely linked to simultaneous political transformations [27,28]. These efforts were dismantled after the 1973 military coup, which reasserted custodial and biomedical models of care and repressed community-based initiatives [29]. Following the return to democracy, Chile implemented a series of national mental health plans that expanded community services and integrated mental health into primary care, achieving significant reductions in long-stay hospitalisation [30,31]. However, this process was embedded within a broader neoliberal model of health system reform, in which policies such as AUGE-GES prioritised cost-effectiveness and standardised interventions, often narrowing the scope of reform and reducing social determinants to individual-level risk factors [27,32].

Taken together, these trajectories show that psychiatric reform unfolds through complex and contested combinations of political mobilisation, institutional experimentation, and technocratic redesign rather than through a single, linear, top-down pathway. Read alongside North American and European experiences, these examples show how different historical relationships between institutional psychiatry, democratisation, social mobilisation, and service reorganisation have produced distinct meanings and pathways of deinstitutionalisation.

In the mid-2000s the “Movement for Global Mental Health” (GMH) pushed for and established a new logic of urgency, prioritisation, and international comparability to mental health policy. This shift was closely tied to the growing influence of the “burden of disease” framework, which uses the Disability-Adjusted Life Year (DALY), a composite measure combining years of life lost (YLL) due to premature mortality and years lived with disability (YLD), to quantify population-level health loss [33]. These metrics made visible the substantial global burden of mental, neurological, and substance-use conditions, and helped establish the “treatment gap”, elevating mental health within global agendas and promoting the “scaling up” of evidence-based interventions in low- and middle-income countries as a moral and technical imperative.

In doing so, they also helped redefine what counted as successful psychiatric reform. Increasingly, progress came to be assessed through indicators such as treatment coverage, workforce expansion, and the delivery of evidence-based interventions rather than through broader questions of institutional transformation. The emphasis on quantification, comparability, and cost-effectiveness privileged forms of knowledge that could be aggregated across contexts, often at the expense of historically and politically grounded understandings of psychiatric reform [34,35]. In this context, the heterogeneous trajectories described above, including those shaped by social movements, democratic transitions, and local intellectual and political traditions, were increasingly folded into a narrative centred on treatment expansion and measurable outcomes [36]. Proponents of this approach often framed the expansion of evidence-based interventions as an urgent matter of equity and human rights, typically emphasising access to psychopharmaceutical and psychosocial treatments [37,38]. While this orientation strengthened global advocacy, it also narrowed the terms through which psychiatric reform was understood, as historically grounded questions of institutional transformation and social relations gradually receded from view [37].

This narrowing has important consequences. The logics of scale and measurement privilege what can be counted and compared, whereas institutional transformation unfolds through slow, contested, and historically embedded processes [39,40]. A sustained engagement with psychiatric reform in South America grounded in social science approaches makes this tension particularly visible, with the region appearing not only as a site of unmet need, as it is often framed within global mental health, but as a space of long-standing intellectual, political, and practical engagement with questions of institutional care, community, and citizenship. The trajectories discussed here embody understandings of deinstitutionalisation that extend beyond the dominant vocabularies of contemporary global mental health, where reform is often framed primarily in terms of scale, coverage, and intervention delivery.

### Institutional persistence and new dilemmas

The narrowing of reform to technocratic terms is difficult to justify in light of persistent asylum-based practices. According to WHO data, as of 2024, only 9% of countries have fully transitioned away from psychiatric hospitals; over half remain in early stages, and 20% have not begun at all [41]. Psychiatric hospitals still account for 62% of inpatient beds and nearly half of all mental health spending, while community services receive just 11%. Involuntary treatment and long-term hospitalisation remain widespread. In many LMICs, psychiatric hospitals are the only available option, and in HICs, closures have not always resulted in rights-based alternatives [42].

Recent advocacy efforts—from WHO and the UN Office of the United Nations High Commissioner for Human Rights to civil society coalitions like United for Global Mental Health—have rightly called for renewed attention to PDI as a global priority [43,44]. The persistence of large psychiatric institutions and coercive practices that violate basic human rights makes such calls both urgent and justified. Yet the question is not only whether PDI should return to the centre of global debate, but on what terms.

Too often contemporary reform agendas frame PDI in technocratic terms: bed reductions, workforce reallocation, legislative compliance, or service coverage. Attention to these metrics and indicators is necessary, but when change is defined predominantly in these terms, the historical trajectories and political and ethical tensions that shape reform become harder to recognise [45,46]. These interpretive limits matter because, after decades of uneven reform, many countries face dilemmas that cannot be resolved—or adequately understood—through scale or legislation alone: community services that reproduce custodial logics [42]; precarity among formal and informal carers [47]; the reduction of care to medicalisation [48]; and persistent stigma and social isolation [49]. These challenges are not simply failures of implementation; they reflect deeper questions about how processes of deinstitutionalisation have been conceptualised and pursued.

PDI therefore remains a political and ethical project whose possibilities also depend on how knowledge about institutions, care and reform is produced. What is required is a richer analytical framework capable of recognising the diversity of reform trajectories, the complexities generated by reform itself, and the unequal conditions under which institutional transformation unfolds.

## Discussion: Re-engaging with deinstitutionalisation

If renewed calls for action are to succeed, they must be grounded in a richer analytical frame. We propose three interconnected lenses for rethinking PDI in global mental health: attending to context and history; decentring the geography of reform; and foregrounding lived experience. Together, these lenses broaden how institutional transformation is understood and how psychiatric reform is analysed across research, policy, professional training, service organisation, and governance.

### 1. Attending to context and history

Re-engaging with PDI begins by recognising that institutional transformation is historically and socially situated. Historical analyses show how the diffusion of a reformist ethos within psychiatry in Europe can be understood as part of a more expansive sociopolitical process linked to the post-World War II period [50]. In parallel, the social sciences have examined how psychiatric institutions operate as sites of power, conflict, and negotiation, deeply intertwined with sociopolitical and cultural processes surrounding madness and care [2,5,6,14,51–55]. These approaches help explain why reforms advance unevenly, how power operates across them, and how custodial logics can persist after legal or policy transformation. PDI is not a technical adjustment achieved through funding or legislation alone; it unfolds across multiple temporalities and through competing professional, political, and legal claims [56].

These dynamics are particularly visible in processes of implementation, where reforms are mediated by existing arrangements, professional identities, and forms of resistance that exceed managerial framings [53,57]. Experiences across diverse settings show that implementation is shaped by factors such as institutional legacies, labour markets, local geographies, and patterns of informal care [9,11,53]. Historical trajectories, whether shaped by colonial systems, authoritarian regimes, or prior waves of reform, continue to influence what is feasible, acceptable, or contested in the present.

This means moving beyond the idea of implementation as a linear translation of policy into practice. Reform unfolds under conditions of uncertainty, often requiring iterative adaptation, negotiation with local actors, and responsiveness to emerging challenges. Strategies such as building community services prior to discharge, engaging local stakeholders in dialogue, or aligning financial incentives with user-centred care illustrate that change depends not only on technical design but on timing, sequencing, and political judgement. Without this perspective, reforms risk reproducing familiar patterns of failure, including transinstitutionalisation, whereby individuals are displaced from psychiatric hospitals into other settings, such as shelters, or highly restrictive residential services, without meaningful social integration or autonomy. Custodial cultures may also persist within ostensibly community-based arrangements, reproducing practices of surveillance, dependency, and segregation in altered forms [11,58,59].

### 2. Decentring the geography of reform

A second requirement is to decentre the geographical assumptions through which psychiatric reform is understood. Much of the literature on psychiatric reform continues to take North Atlantic experiences as its principal point of reference [45]. Even where these trajectories are approached critically, particularly in relation to the failures of dehospitalisation in the United States [60], they often shape the terms through which deinstitutionalisation is understood [4]. However, reform trajectories in South America and other regions have been shaped by grassroots mobilisation, democratic transition, and struggles for social rights [61,62]. Such trajectories complicate linear accounts of PDI, expanding the conceptual horizons of reform and enabling dialogue and learning across settings.

A large proportion of contemporary global mental health research and policy guidance is directed towards low- and middle-income countries [63,64]. However, these settings are often approached primarily through the language of resource scarcity, treatment gaps, and service expansion, and not through the histories and political struggles that have shaped their mental health systems [65,66]. Reforms are frequently understood in technical terms, while the social movements, institutional conflicts, and locally grounded traditions that have informed psychiatric transformation in many contexts remain marginal. This narrowing not only affects how problems are diagnosed, but also what kinds of reforms become visible, legitimate, or transferable within global mental health.

This has implications for how understandings of reform circulate in global mental health. Funding priorities, publication practices, and professional training have tended to privilege models that travel easily across contexts, often sidelining experiences rooted in different institutional histories or political conditions [67]. Experiences from Eastern Europe, Latin America, Africa, and Asia instead show that PDI often advances through context-specific combinations of legal change, service innovation, and political negotiation [3,68]. Recognising this diversity expands what can count as psychiatric reform and reopens institutional transformation as a historically situated process rather than a technical programme of implementation.

### 3. Foregrounding lived experience

Re-engaging with PDI also requires recognising lived experience as a source of knowledge about institutional transformation. Users and survivors have demanded inclusion within existing debates while also producing important counter-histories and critical analyses of deinstitutionalisation itself [69]. Survivor scholarship and activism have documented how transitions to community care frequently unfolded through abandonment, poverty, homelessness, fragmented services, and new forms of coercion [70]. These developments reflect a wider shift in mental health policy and research towards recognising lived experience as a source of conceptual and historical knowledge in its own right [71]. Yet the knowledge, experience, expectations, and concerns of users, survivors, families, and frontline workers remain rarely centred in policy debates on PDI.

These developments challenge the tendency to frame lived experience primarily as a source of “subjective” testimony accompanying professionally authorised knowledge. As Britz and Jones argue, psychiatry and mental health policy have historically positioned users and survivors as objects of intervention and not as theorists, historians, and producers of knowledge in their own right [70]. Re-engaging with PDI involves recognising experiential knowledge as a source of alternative interpretations of institutionalisation, community, care, coercion, and recovery [59,70].

Across different settings, people with lived experience have described community life as shaped simultaneously by new possibilities for autonomy and by persistent precarity, stigma, bureaucratic fragmentation, and custodial logics reproduced in dispersed forms. Survivor-led initiatives have also demonstrated alternative ways of organising support, care, and community beyond paternalistic models of service delivery [69,70,72]. Inclusion requires forms of shared governance in which people with lived experience and those directly involved in care have a visible role in shaping priorities, monitoring processes, and evaluating outcomes [73]. Across different contexts, this has taken the form of user representation in governance structures, peer-led services, involvement in workforce training, and participation in recruitment, evaluation, and research. Such arrangements move beyond consultation towards redistribution of authority, recognising lived experience as a form of expertise with implications for how services are designed, delivered, and historically understood.

Re-engaging with PDI therefore involves taking seriously the epistemic transformation brought about by lived experience, recognising users and survivors as producers of knowledge about institutionalisation, community, care, and recovery [74]. Recognising lived experience in this way expands not only participation in reform but also how institutional transformation itself is understood, governed, and evaluated. Taken together, these three lenses suggest that psychiatric reform is best understood as an uneven process of institutional transformation shaped by history, context and competing forms of knowledge. Reform therefore depends not only on technical design but on how institutions, authority, and care are negotiated across settings.

## Conclusion

PDI is not a closed chapter but a shifting terrain of struggle. The persistence of asylum-based practices, alongside the emergence of new dilemmas, reflects not only gaps in implementation but the limits of how reform has been framed and pursued. As long as reform is understood primarily through technocratic metrics such as beds, coverage, laws and workforce, it will remain difficult to grasp, and address, the transformations it demands.

This is precisely why deinstitutionalisation still matters. Psychiatric institutions have not disappeared; they have persisted, mutated, and, in some contexts, re-emerged through new arrangements of care, coercion, abandonment, and social control. At the same time, decades of uneven reform have generated new contradictions: transinstitutionalisation, fragmented systems of care, the reproduction of custodial logics within community settings, and persistent inequalities in who can access autonomy, support, and citizenship. These challenges cannot be understood through implementation gaps alone. They raise deeper questions about how institutional transformation is imagined, organised, and evaluated. Although contemporary agendas increasingly invoke the language of mental health reform or system transformation, these shifts in terminology do not, by themselves, address the political, ethical and institutional questions raised by deinstitutionalisation. Rather, they risk obscuring the continuing relevance of institutional power, coercion, and citizenship as central concerns of reform.

Re-engaging with PDI therefore calls for a reorientation in how reform is understood and pursued across the multiple sites where it is enacted: research, policy, legal systems, professional training, activism, and everyday practices of care. This includes recognising the diversity of reform trajectories, drawing on intellectual traditions beyond dominant North Atlantic frameworks, and taking seriously the epistemic contributions of lived experience to understanding institutional transformation.

Reclaiming PDI as a political, ethical, and epistemological project is not a matter of returning to past radicalism, but of recovering a richer understanding of institutional transformation—one informed by diverse reform trajectories and open to the forms of knowledge generated through lived experience. Only from that perspective can contemporary mental health reform engage seriously with the enduring questions that processes of deinstitutionalisation continue to pose [45,59,70].
